# Estrogen receptor alpha regulates the expression of adipogenic genes genetically and epigenetically in rat bone marrow-derived mesenchymal stem cells

**DOI:** 10.7717/peerj.12071

**Published:** 2021-09-10

**Authors:** Ceylan V. Bitirim, Zeynep B. Ozer, Kamil C. Akcali

**Affiliations:** 1 Stem Cell Institute, Ankara University, Ankara, Turkey; 2 Department of Biophysics, Faculty of Medicine, Ankara University, Ankara, Turkey

**Keywords:** Estrogen receptor, Epigenetic, Mesenchymal stem cells, Obesity, Adipogenesis, Molecular biology, genetics

## Abstract

Regulation of the efficacy of epigenetic modifiers is regarded as an important control mechanism in the determination and differentiation of stem cell fate. Studies are showing that the effect of estrogen is important in the differentiation of mesenchymal stem cells (MSCs) into adipocytes, osteocytes, and chondrocytes. Activation of certain transcription factors and epigenetic modifications in related genes play an active role in the initiation and completion of adipogenic differentiation. Understanding the role of estrogen in diseases such as obesity, which increases with the onset of menopause, will pave the way for more effective use of estrogen as a therapeutic option. Demonstration of the differentiation tendencies of MSCs change in the presence/absence of estrogen, especially the evaluation of reversible epigenetic changes, will provide valuable information for clinical applications. In this study, the effect of estrogen on the expression of genes involved in adipogenic differentiation of MSCs and accompanying epigenetic modifications was investigated. Our results showed that estrogen affects the expression of adipogenesis-related transcription factors such as PPARy, C/EBPα and Adipsin. In addition, after estrogen treatment, increased accumulation of estrogen receptor alpha (ERα) and repressive epigenetic markers such as H3K27me2 and H3K27me3 were observed on the promoter of given transcription factors. By using co-immunoprecipitation experiments we were also able to show that ERα physically interacts with the zeste homolog 2 (EZH2) H3K27 methyltransferase in MSCs. We propose that the increase of H3K27me2 and H3K27me3 markers on adipogenic genes upon estrogen treatment may be mediated by the direct interaction of ERα and EZH2. Taken together, these findings suggest that estrogen has a role as an epigenetic switcher in the regulation of H3K27 methylation leading to suppression of adipogenic differentiation of MSC.

## Introduction

Mesenchymal stem cells (MSC) are adult stem cells and give rise to adipocytes, osteocytes, and chondrocytes. They tend to differentiate into one cell line while suppressing differentiation into another cell type (Jeong & Yoon, 2011). Due to their wide-range differentiation capacity, MSCs are widely used in clinical and preclinical studies. After menopause, the decrease in circulating estrogen causes an increase in adipose tissue (Heim et al., 2004; Heine et al., 2000; Stubbins et al., 2012; Jeong & Yoon, 2011). Moreover, estrogen replacement therapy can reverse the enhanced fatty acid formation resulted from estrogen deficiency in postmenopausal women (Jeong & Yoon, 2007; Carani et al., 1997). Adipogenesis is a well-orchestrated and complex multistep physiological process that requires molecularly sequential regulation of transcription factors such as CCAAT/enhancer-binding protein alpha (C/EBPα), PPAR-γ, and Adipsin. Among these factors, PPAR-γ and C/EBPα are key regulators. Through RNA sequencing, it was demonstrated that co-expression of PPARγ and C/EBPα leads to synergistic activation of many key metabolic adipocyte genes (Madsen et al., 2014; Tang, Zhang & Daniel Lane, 2004; Musri et al., 2010; Zuo, Qiang & Farmer, 2006). In particular, PPARγ plays a dominant role in this process (Meyer et al., 2016). Adipsin, which is also regulated by PPARγ, is regarded as a late marker of adipocyte differentiation. Importantly, adipsin deficiency directly modulates BMSC fate by hindering their differentiation into adipocytes (Aaron et al., 2021). Expression of early and late adipogenic differentiation markers in MSCs can be inhibited by treatment of both estrogen and phytoestrogen (Evans, Barish & Wang, 2004; Kim et al., 2009). There is an inverse correlation between osteogenesis, chondrogenesis, and adipogenesis in MSCs (Hong et al., 2011; Zhao et al., 2011; Okazaki et al., 2002). Estrogen plays an important role in the reciprocal control of adipogenic and osteogenic differentiation in progenitor cells (Chen et al., 2016; Dang & Löwik, 2004; Foryst-Ludwig et al., 2008; Jeong & Yoon, 2011; Hong, Colpan & Peptan, 2006). The observation of an increase in body fat percentage and the incidence of obesity with menopause and ageing has been attributed to deregulation in progenitor cell differentiation. This may explain the clinical effects of decreased circulating estrogen, however, the molecular and epigenetic mechanisms related to this effect are still elusive.

ERα and ERβ work with epigenetic mediators such as histone acetyltransferases (HATs), histone deacetylase (HDACs), and SWI/SNF complexes, enabling estrogen to play a role in epigenetic regulation of the transcription of target genes. (Hong et al., 2011; Zhao et al., 2011; Stossi, Madak-erdogan & Benita, 2009; Zubairy & Oesterreich, 2005). Estrogen both repress and stimulate the gene expression through estrogen receptors. Epigenetic mediators, zest homolog 2 (EZH2) and histone deacetylase 9c (HDAC9c) have been shown to play a role in the regulation of age-related osteogenic and adipogenic differentiation processes in MSCs (Chen et al., 2016; Chen et al., 2011). EZH2 is considered one of the key epigenetic regulators in development and cell differentiation. EZH2 is one of the core subunits of polycomb repressive complex 2 (PRC2) and catalyzes mono-, di-, and trimethylation of histone three lysine 27 (H3K27), which are epigenetic hallmarks of repressed chromatin (Mu et al., 2018). Similar to estrogen, previous studies demonstrated that EZH2 plays an important role in the regulation of MSC differentiation. It has been reported that EZH2 has a pivotal role in the adipogenic lineage commitment of MSCs in an age-dependent manner (Chen et al., 2016). Genome-wide profiling studies have revealed that EZH2 may act as a molecular switcher to adjust the balance between osteogenic and adipogenic differentiation in MSCs (Hemming et al., 2016; Hemming et al., 2014; Chen et al., 2016; Wang et al., 2010). However, the role of EZH2 in the molecular mechanism of the coordination of the adipogenic differentiation and the estrogen-mediated epigenetic control in MSC differentiation is not elucidated. Here, we aimed to further elucidate the association between estrogen and epigenetic modifications including histone and DNA methylation which underlie the regulation of MSC differentiation into adipocytes. We propose that the physical interaction between EZH2 and ERα in the presence of estrogen may be the epigenetic mechanism underlying the transcriptional suppression of key adipogenic genes such as PPARy, C/EBPα and Adipsin. Our data support that estrogen inhibits adipogenesis by forming a co-repressor complex that acts on adipogenic genes with epigenetic regulators.

## Materials & methods

### Animal preparation and cell culture

Heterogeneous bone marrow cells were isolated from the tibia and femur of ten normal females and ten ovariectomized 9-week-old, 280–300 g Sprague–Dawley rats. Ovariectomization procedure was performed to mimic the post-menopausal stage. The ovariectomy procedure was performed under full anaesthesia using ketamine at a concentration of 30 mg/kg. Almost after 2 months after the operation, the ovariectomized animals were used for experiments to ensure the total deficiency of estrogen. The animals that were showing signs of sickness and lost more than 20% weight after the operation were excluded. The animals were always kept and permitted unlimited access to food and water. The animals express their normal behavioural patterns, since they have sufficient space and proper facilities. The behavioural and physiological changes of ovariectomized animals were monitored daily by the veterinarian. They were housed under controlled environmental conditions in the animal holding facility of the Department of Molecular Biology and Genetics at the İhsan Doğramacı Bilkent University (Ankara, Turkey). The experimental protocol is arranged with İhsan Doğramacı Bilkent University’s guidelines on humane care and the use of laboratory animals. All experimental protocols were approved by the Laboratory Animal Welfare and Ethics Committee of Bilkent University. Permission number for using animals: 2009/18.

After sacrification, bone marrow cells were flushed with a five mL syringe in DMEM (HyClone, Logan, UT, USA) containing 10% fetal bovine serum (FBS) (HyClone, Logan, UT, USA) and 1% penicillin-streptomycin antibiotic (HyClone, Logan, UT, USA) after cervical dislocation. The bone marrow cells were cultured in MesenCult medium (Stem Cell Technologies, Vancouver, Canada) including 20% supplement (Stem Cell Technologies, Vancouver, Canada) and 1% penicillin-streptomycin solution (HyClone, Logan, UT, USA) for 14 days. After washing with sterile 1XPBS the media were changed every 4 days. In addition to the untreated control group, the cells were treated 10^−7^ M estrogen (17 ß-estradiol, Sigma, Schnelldorf, Germany) and 10^−6^ M tamoxifen (Sigma, Schnelldorf, Germany) from the 1st day of culture until the 14^th^ day to obtain the estrogen-treated and tamoxifen-treated groups.

### Quantitative RT-PCR

Total RNA isolation from MSCs at the end of the 14th day of the cell culture was performed using Nucleospin RNA II kit isolation kit (Macherey-Nagel, Düren, Germany) according to instructions of the manufacturer. For cDNA synthesis, one μg of RNA was used for each reaction. cDNA synthesis was performed using DyNAmo cDNA synthesis kit (Finnzymes, Espoo, Finland). cDNA was amplified by SYBR green real-time PCR kit (Finnzymes, Espoo, Finland). Expression of transcription factor genes was normalized according to cyclophilin expression level. Fold changes in the expression of the genes were analyzed based on the comparative (2^−ΔΔCt^) method. As a calibrator, the normal female MSC group was used. The primer sequences used in this study are listed in Table 1.

**Table 1:** The primers used for qRT-PCR and ChIP-PCR.

Genes	Sequence	Product length
C/EBPα	Forward: 5′-AGTCGGTGGATAAGAACAGC-3’ Reverse: 5′-CATTGTCACTGGTCAACTCC-3’	138
PPARγ	Forward: 5′-TTTTCAAGGGTGCCAGTTTC-3′ Reverse: 5′-AATCCTTGGCCCTCTGAGAT-3′	198
Adipsin	Forward: 5′-TGGTGGATGAGCAGTGGGT-3′ Reverse: 5′-AGGGTTCAGGACTGGACAGG-3′	110
RUNX2	Forward: 5′-TAACGGTCTTCACAAATCCTC-3′ Reverse: 5′-GGCGGTCAGAGAACAAACTA-3′	115
Cyclophilin	Forward: 5′-GGGAAGGTGAAAGAAGGCAT-3′ Reverse: 5′-GAGAGCAGAGATTACAGGGT-3′	211

### Co-Immunoprecipitation (Co-IP) assay and western blotting

MSCs were scraped from cell culture flasks in ten ml 1XPBS with a scraper. The cell suspension was centrifuged at 900 $\times$ g for 5 min at 4 °C. The pellet was dissolved in lysis buffer and incubated on ice for 30 min by finger tipping every 5 min. Then, the lysates were centrifuged at 12,000 $\times$ g for 20 min at 4 °C. The supernatant containing total proteins was taken and stored at −80 °C. The protein concentration was determined by performing BCA assay (Thermo, Rockford, IL, USA). Protein lysates were immunoprecipitated using anti-ERα (cat.no: sc-8002; Santa Cruz, CA, USA), monoclonal antibody and IgG (cat.no: sc-2025; Santa Cruz, CA, USA) monoclonal antibody as a negative control. Precipitation was performed using protein G magnetic beads of Co-IP kit (cat.no: 88847; Thermo, Rockford, IL USA) and the procedure was performed according to the manufacturer’s protocol. The protein lysates were separated by 10% SDS-PAGE and transferred to a polyvinylidene fluoride membrane. The membranes were blocked in 5% BSA in 0.1% TBS-T. Immunoblotting was performed using the monoclonal antibody against EZH2 (cat.no: 5246; 1:500; Cell Signaling, Danvers, MA, USA). Membranes were incubated in antibody solution at the concentration for overnight and 4 °C. The secondary antibody anti-Rabbit-HRP (cat.no: 7074; 1:5,000; Cell Signaling, Danvers, MA, USA) was applied for 1 h in blocking solution. Finally, Super Signal West Femto Maximum Sensitivity Substrate (Thermo Scientific, Waltham, IL, USA) was used for chemiluminescence detection. The membranes were visualized by ChemiDoc gel imaging system (Bio-Rad, Hercules, CA, USA).

### Chromatin Immunoprecipitation (ChIP) assay

Simple-ChIP Enzymatic Chromatin IP Kit (Agarose Beads) (Cell Signaling Technology, Danvers, MA, USA) was used for ChIP assay according to the manufacturer’s protocol. MSCs were incubated with or without either estrogen. A total of 9 μg of chromatin DNA was immunoprecipitated using anti-ERα (cat.no: sc-8002; Santa Cruz, CA, USA), monoclonal antibody, anti-H3K27me2 monoclonal antibody (cat.no: 9728; Cell Signaling, Danvers, MA, USA), and anti-H3K27me3 monoclonal antibody (cat.no: 9733; Cell Signaling, Danvers, MA, USA) at 1:50 dilution rate. IgG (cat.no: sc-2025; Santa Cruz, CA, USA) monoclonal antibody was used as a negative control. A total of 10% of DNA was used as input. ChIP assay was analyzed with quantitative PCR using qPCR 2X Master mix (Ampliqon, Denmark) and results were expressed using the percent input method: Percent input = (Primer Efficiency) ^ (CT input – CT IP sample) $\times$ Percentage of input. qPCR conditions were 95 °C for 15 min, 95 °C for 30 s, 60 °C for 1 min, 72 °C for 1 min and 30 cycles, 72 °C for 10 min.

### Bisulfite sequencing

DNA methylation status on the promoter of PPARγ and C/EBPα genes was analyzed by bisulfite sequencing. Either DNeasy Blood & Tissue Kit (Qiagen, Germantown, MD, USA) or NucleoSpin Tissue kit (Machery-Nagel, Düren, Germany) was used for DNA purification following the manufacturer’s instructions. 350 ng of DNA was used for each bisulfite conversion reaction performed using EpiTech Bisulfite Kit (Qiagen, Germantown, MD, USA). Amplification of converted DNA was performed by HotstarTaq polymerase (Qiagen, Germantown, MD, USA). Bisulfite-specific primers were created using MethPrimer software (Li & Dahiya, 2002) for PPAR γ and C/EBPα. ERα primers were designed as given previously (Zama & Uzumcu, 2009). The primer sequences used in bisulfite sequencing experiments are listed in Table 2. PCR products were extracted from 1% agarose gel using QIAquick Gel Extraction Kit (Qiagen, Germantown, MD, USA) and cloned into DH5α bacteria cells by TA Cloning (pGEM-T Easy Vector system, Promega, Madison, WI, USA). Plasmid DNA was isolated by using Plasmid Mini Kit (Qiagen, Germantown, MD, USA) and clones were sequenced by commercial services (İONTEK, Ankara, Turkey; DONE Genetics, Ankara, Turkey).

**Table 2:** The primers used for Bisulfite Specific PCR.

Genes	Sequence		Product length
C/EBPα	Forward	5′TGGGTGTTTATTAGGTTTTTTTTGT-3′	167
	Reverse	5′-AACCCCCTATCCAATCCTTAAA-3′	
PPARγ	Forward	5′TTTTAGGTTTTTTTAGAAGGTGTTT-3′	368
	Reverse	5′TCCAATTAACCCTACCCTATACTC-3′	

### Statistical analysis

The statistical significance between the groups of qRT-PCR and western blotting experiments were determined by the unpaired Student’s t-test. The values from qRT-PCR were expressed as the means ± SD (*N* = 6) and from ChIP-qPCR were expressed as the means ± SD (*N* = 3) and the measurements from western blotting were expressed as the means ± SD (*N* = 3). *p* < 0.05 was chosen statistically significant. *N* number indicated the number of rats each of which was used as single donor.

## Results

### Transcriptional repression of adipogenic genes upon estrogen treatment in MSC

**Figure 1:**
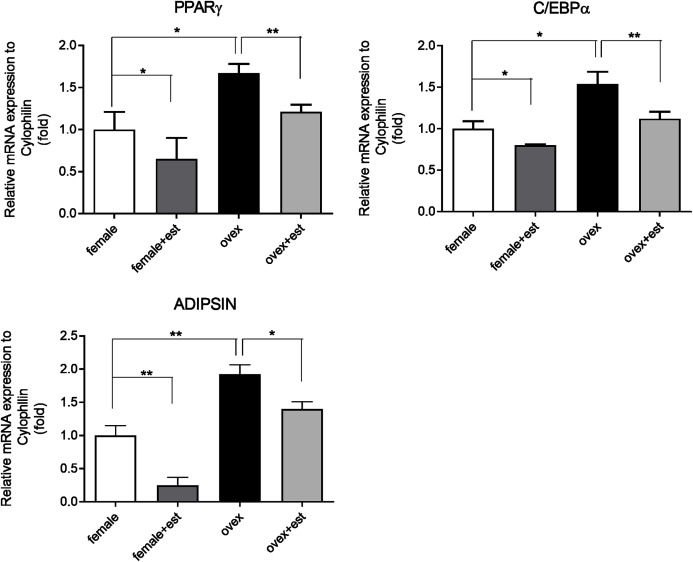
Changes in mRNA expression of the adipogenic transcription factors upon estrogen treatment in MSCs. The mRNA expression levels of adipogenic genes (A) PPARγ, (B) C/EBPα, (C) Adipsin were measured by qRT-PCR in MSC. MSCs isolated from normal (female) and ovariectomized female (ovex) rats were cultured in the absence and presence of estrogen. Transcript levels were normalized to Cylclophilin. * indicates *p* < 0.05, ** indicates *p* < 0.01. All data are represented as the mean ± SD (*n*= 6).

To determine whether the expression of adipogenic genes changes in the presence of estrogen, we first analyzed the transcription levels of key adipogenic genes; PPARγ, C/EBPα, and Adipsin in MSCs isolated from normal females (female) and ovariectomized rats (ovex). MSCs were incubated for 14 days in the presence and absence of estrogen. Previously, the inhibitory effect of estrogen on the expression of adipogenic differentiation markers in MSCs was reported (Zhao et al., 2011). According to our results, estrogen treatment caused significant downregulation of PPARγ (Fig. 1A), C/EBPα (Fig. 1B) and Adipsin (Fig. 1C) in MSC obtained from both normal female and ovex rat. In addition to this, estrogen deficiency results in increased mRNA expression of PPARγ, C/EBPα, and Adipsin. The transcript levels of the major regulators of adipogenesis are significantly higher in ovex-MSCs compared to normal female-MSCs, suggesting that estrogen deficiency may associate with the adipogenic commitment of MSCs.

### Physical interaction of ERα with EZH2

**Figure 2:**
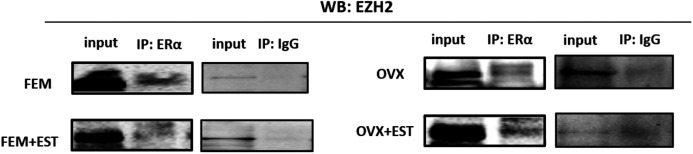
Protein-protein interaction between EZH2 and ERα. Protein-protein interaction between EZH2 and ERα was examined by CoIP assay. MSCs isolated from normal female and ovex rats were cultured in the absence and presence of estrogen. MSC protein lysates were immunoprecipitated with anti-ERα and anti-IgG antibodies followed by imunoblotting with anti-EZH2 antibody. WB, western blott; IP, immunoprecipitation; FEM,: female; OVX, ovex.

To address the epigenetic mechanism underlying the suppressive effect of estrogen on the adipogenic genes, firstly we investigate the functional link between ERα and EZH2 and analyzed whether the two proteins could interact. EZH2 catalyzes methylation of H3K27 which is a histone modification associated with gene repression. To address this issue, the co-IP assay was performed. The MSCs isolated from normal female and ovex animals were incubated with estrogen. Immunoprecipitation was performed in the MSC protein extract using ERα antibody followed by blotting with anti-EZH2 antibody. The direct interaction between ERα and EZH2 both in normal and ovex MSC was indicated (Fig. 2). Notably, the ERα-EZH2 interaction tends to be induced strongly upon estrogen treatment in ovex group. In the light of these observations, we strongly suggest that estrogen-dependent adipogenic gene regulation might occur through the direct interaction of ERα with EZH2 to mediate the H3K27 methylation on the promoter of adipogenic genes. Moreover, estrogen administration may possess a much stronger effect on gene expression and differentiation in ovex-MSCs than normal female-MSCs.

### Binding of ERα and methylated H3K27 on adipogenic genes upon estrogen treatment

**Figure 3:**
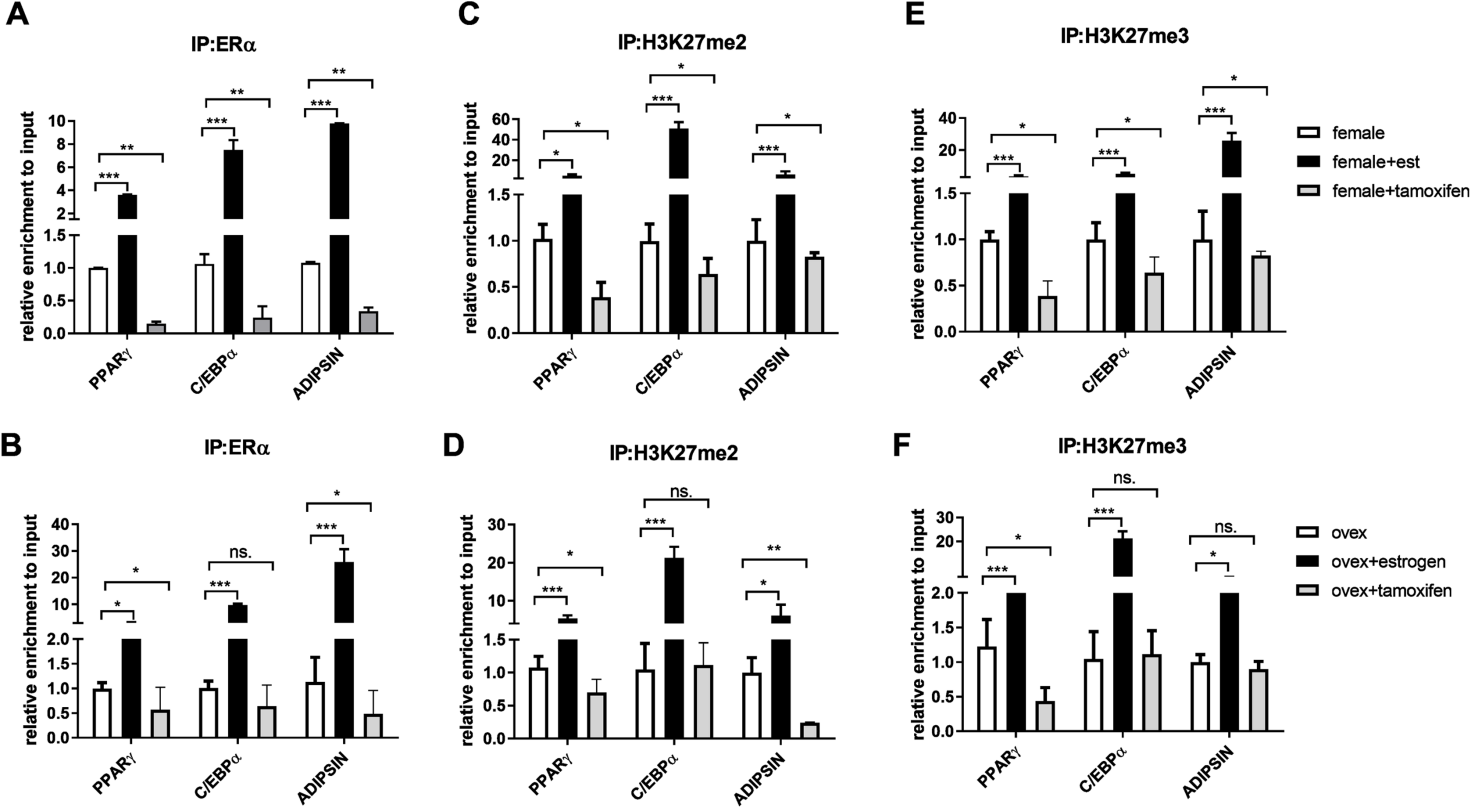
Effects of estrogen on the accumulation of ERα and methylated histone proteins to the promoters of adipogenic transcription factor genes. The enrichment of (A, B) ERα, (C, D) H3K27me2 and (E, F) H3K27me3 on PPARγ, C/EBPα and Adipsin genes were analyzed by ChIP analysis. Genomic DNA extracted from normal female and ovex female rat MSCs which were cultured either estrogen or tamoxifen were immunoprecipitated with anti-ERα, anti-H3K27me2 and anti-H3K27me3. The alteration in the accumulation of these proteins on adipgogenic genes was quantified by qPCR. The data were expressed as a percent of input. An asterisk (*) indicates *p* < 0.05, ** indicates *p* < 0.01, *** indicates *p* < 0.001. All data are represented as the mean ± SD (*n* = 3).

After indicating the direct interaction between ERα and EZH2, to further understand the cross-talk between ERα and histone modifications, we next examined whether ERα regulates the methylation status of H3K27 in an estrogen-dependent manner. To test this possibility, we performed a conventional ChIP-qPCR assay to analyze the binding status of ERα, H3K27me2, and H3K27me3 on PPARγ, C/EBPα, and Adipsin genes. In ChIP assay, Tamoxifen was also used as an antagonist to ERα to validate the effect of estrogen on binding of ERα and methylated H3K27. MSCs isolated from normal female and ovex rats were incubated either with estrogen or tamoxifen. We have shown that estrogen treatment induces almost three fold in the binding of ERα on PPARγ gene, and almost tenfold on C/EBPα in MSCs isolated from both normal (Fig. 3A) and ovex rat (Fig. 3B). In particular, estrogen treatment stimulated the binding of ERα to Adipsin almost ten fold in female group and 25 fold in ovex group. This result suggests that ERα is directly involved in adipogenic gene expression *via* binding on PPARγ, C/EBPα, and Adipsin genes in MSCs. The enhancement in the occupancies of H3K27me2 and H3K27me3 after estrogen treatment on the adipogenic genes was also observed (Figs. 3C–3F). The existence of H3K27me2 induced by estrogen more than two fold on PPARγ and more than five fold on Adipsin both in female and ovex MSCs. The accumulation of H3K27me2 on C/EBPα was dramatically reached up to 40 fold in female and up to 20 fold in ovex (Figs. 3C–3D). Similar to H3K27me2, estrogen treatment also increased the H3K27me3 binding more than three fold on PPARγ and more than five fold on C/EBPα and Adipsin genes (Figs. 3E–3F). Especially the accumulation level reached up to 20 fold on Adipsin and C/EBPα in female and ovex groups, respectively. Tamoxifen treatment significantly decreased the enrichment of ERα, H3K27me2 and H3K27me3 on adipogenic genes in female groups (Figs. 3A–3E). On the other hand, in the ovex groups, we did not observe this significant reduction of H3K27me2 and H3K27me3 on C/EBPα and Adipsin genes (Figs. 3D–3F). Taken together, our data demonstrated that the downregulation of transcription of PPARγ, C/EBPα, and Adipsin may be correlated with the increased recruitment of H3K27me2 and H3K27me3 on the promoter regions.

### CpG methylation status in the promoter regions of adipogenic transcription factors

**Figure 4:**
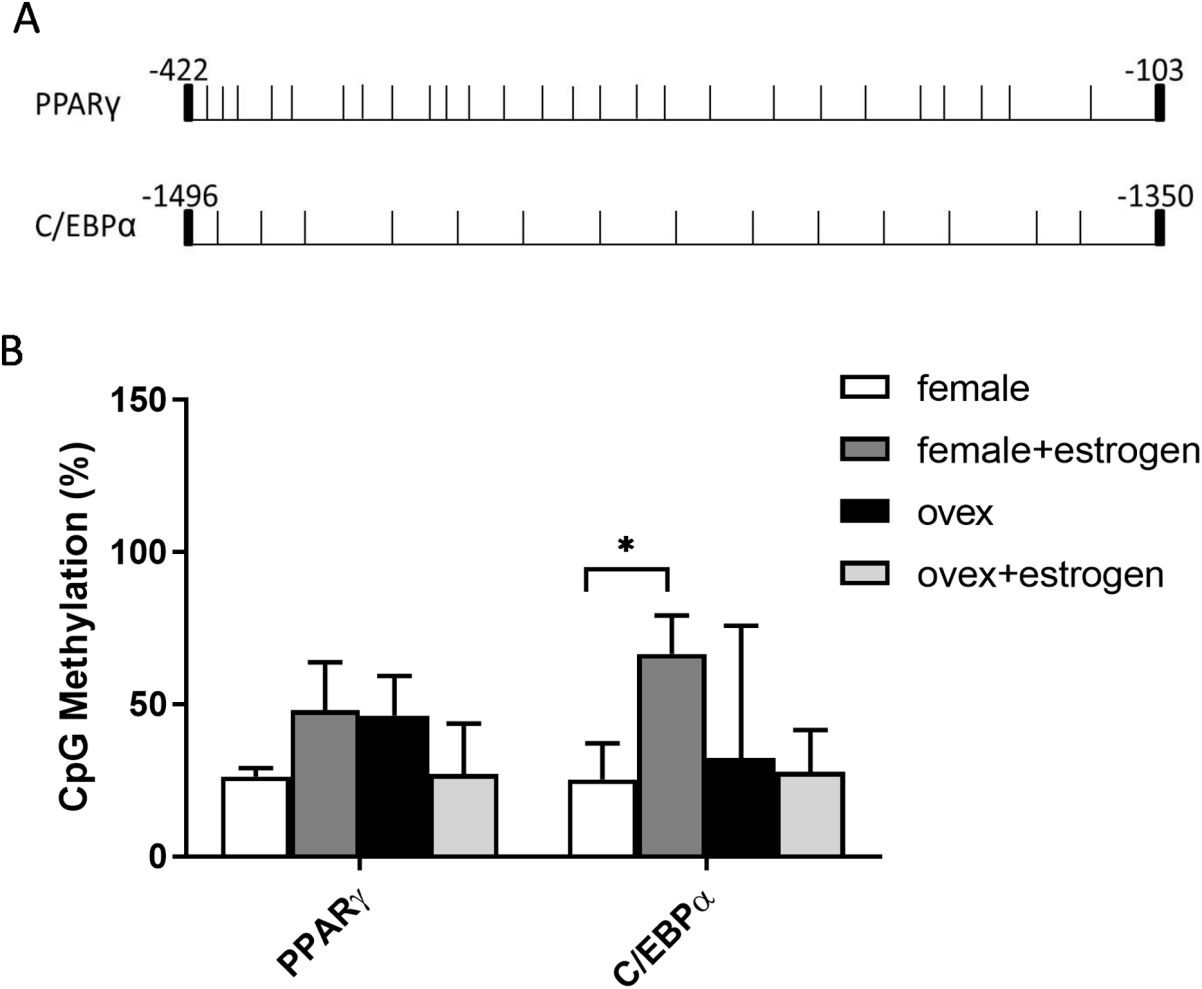
Determination of the level of CpG methylations at the promoters of PPARγ and C/EBPα by bisulfite sequencing analysis in MSCs. (A) Distrubition of CpG residues. Each vertical line represents a single CpG residue. (B) Bisulfite analysis of PPARγ and C/EBPα from 5-7 MSC clones from normal female and ovex female rat. Data shows the percentage of global CpG methylation. Bisulfite converted DNA was amplified with bisulfite specific primers for PPARγ, C/EBPα and ERα. Average percentage of CpG methylation was calculated. As asterisk (*) indicates *p* < 0.05. All data are represented as the mean ± SD.

In addition to histone methylation, we next tested the other major epigenetic modification, DNA methylation. CpG methylation of two master regulator genes; PPARγ and C/EBPα, in polyclonal cultures of MSCs, were analyzed by bisulfite DNA sequencing. PPARγ and C/EBPα are major contributors to the expression of adipogenesis-related gene Adipsin (Rosen et al., 2002). The distribution of CpG residues in each promoter region, which are given in Fig. 4A, was examined and the data were expressed as a percentage of CpG methylation for both PPARγ and C/EBPα (Fig. 4B). Despite some heterogeneity between clones, the level of CpG methylation at each gene was determined taking the average percentage of CpG methylation from 5–7 bacterial colonies. Although DNA hypermethylation on C/EBPα was significant upon estrogen in normal female-MSCs, PPARγ displayed not quite significantly higher average methylation in the estrogen-treated group compared to the untreated group in normal females. On the contrary, there were no significant differences observed within ovex groups. Notably, estrogen deficiency did not result in a significant change of DNA methylation status between ovex and normal female-MSCs.

## Discussion

Epigenetic mechanisms are reversible, which is why the identification of genetic and epigenetic mechanisms underlying reprogramming of key aspects of MSC such as cell fate determination may provide important insight for clinical applications in treatments of obesity (Ling & Rönn, 2019). Estrogen deficiency is considered a major cause of osteoporosis, osteoarthritis, and obesity in postmenopausal women (Palmisano, Zhu & Stafford, 2017; Evans, Barish & Wang, 2004; Zhao et al., 2008). Prior studies were demonstrated that estrogen represses adipogenesis by inhibiting the expression of early and late adipogenic differentiation markers while promoting osteogenesis in MSCs (Glenske et al., 2019; Takada et al., 2007; Ye et al., 2012; Sävendahl, 2005; Zhao et al., 2011; Zhang et al., 2012; Zhou et al., 2001). Moreover, the ChIP-seq analysis has shown that transitions of epigenetic marks, such as H3K9ac, H4K5ac, H3K4me1, H3K4me3, and H3K36me3 on transcription factors involved in MSC differentiation, such as Runx2, C/EBPβ, C/EBPα, PPARγ coordinate the cell fate decision of MSC (Meyer et al., 2016).

Here, we investigated how estrogen and ERα coordinate transcriptional regulation of adipogenic genes in MSCs both at the genetic and epigenetic levels.

PPARγ and C/EBPα lead to synergistic activation of many key metabolic adipocyte genes (Madsen et al., 2014). In particular, PPARγ plays a dominant role in this process. Due to these reasons, we have chosen PPARγ and C/EBPα as early and essential adipogenesis markers. In addition, late adipocyte marker Adipsin is also regulated by PPARγ and is essential for MSC differentiation (Aaron et al., 2021).

Our data revealed that estrogen downregulates mRNA expression of major adipogenic genes; PPARγ, C/EBPα, and Adipsin, while estrogen deficiency induces the expression of these genes in MSCs. Thus, estrogen treatment reverses this induced effect caused by a lack of estrogen suppressing gene transcription. These results agree with observations that report estrogen ERα dependent regulation of adipose tissue formation and decrease in body fat distribution *via* estrogen replacement in early postmenopausal women (Babaei et al., 2017; Pedersen et al., 1992; Dang & Löwik, 2004). Moreover, within the mesenchymal lineage, RUNX2 and PPARγ are major factors that are responsible for osteogenic and adipogenic differentiation, respectively (Meyer et al., 2016). To verify the specific effect of estrogen on adipogenic-related genes independently of other possible factors, we evaluated the expression level of osteoblast related gene Runx2. Our data demonstrated that estrogen treatment causes a significant increase in the expression of Runx2 while suppressing expression of adipogenesis related genes (Fig. S1).

To support our genetic data, we aimed to delineate the potential effects of estrogen hormone and ERα in the epigenetic regulation mechanisms which control the adipogenesis in MSCs. It suggests that many co-regulators and transcription factors possessing chromatin-modifying activities maintain epigenetic regulation during adipogenesis (Teven et al., 2011). The histone modifications play important role in changing chromatin architecture upon adipogenic differentiation.

According to previous studies, various histone modifications are crucial for chromatin reorganization and regulation of gene transcription during MSC adipogenic differentiation. The transcriptional control of PPARγ and CEBP/α through the formation of H3K4/H3K9me3 bivalent domains in lineage-committed mesenchymal stem cells and preadipocytes was also established (Matsumura et al., 2015; Li et al., 2010). To establish the link between the presence of estrogen and the modulation of inactive histone proteins on adipogenic gene promoters, we firstly examined the binding status of ERα on these genes. ERα is a nuclear receptor and acts as a transcription factor. Co-activators and co-repressors are directly recruited by nuclear receptors as a consequence of ligand-binding (Liu, Zou & Qin, 2017; Stossi, Madak-erdogan & Benita, 2009; Shang et al., 2000). In contrast to the well-known stimulatory effect of estrogen, ligand-activated ERα also represses gene expression by recruiting co-repressor/histone deacetylase (HDAC) or histone methyltransferase-containing complexes (Stossi, Madak-erdogan & Benita, 2009). In this study, we reported the enhanced recruitment of ERα to the PPARγ, C/EBPα, and Adipsin upon estrogen treatment. ERα works highly dynamically as a nuclear receptor for a cohort of co-regulator complexes such as p160, p300, and CtBP1 and enzymatic complexes such as SWI/SNF, histone acetyltransferases (Shang et al., 2000; Senyuk, Sinha & Nucifora, 2005; Stossi, Madak-erdogan & Benita, 2009). Stossi and colleagues reported that ERα initiates transient stimulation of transcription and it uses p300 to recruit CtBP1-containing complexes. These complexes modulate chromatin modifications which lead to transcriptional repression at early estrogen-repressed genes (Stossi, Madak-erdogan & Benita, 2009). Like ERα binding to adipogenic genes, estrogen treatment dramatically induces enrichment of methylated H3K27 on the PPARγ, CEBP/α, and Adipsin in MSCs, indicating that binding of liganded-ERα on these promoters enhance methylation of H3K27. These findings support our qRT-PCR data suggesting that estrogen may suppress adipogenic gene expression through accumulation of methylated H3K27 proteins on the gene promoters. In addition, we have demonstrated that tamoxifen treatment drastically decreases the accumulation of ERα, H3K27me2 and H3K27me3 on PPARγ, C/EBPα and Adipsin which is in support of epigenetic repression is mediated by ERα signaling. On the other hand, we did not observe this significant reduction of H3K27me2 and H3K27me3 on C/EBPα and Adipsin genes in the ovex groups which lack estrogen. These results suggest that estrogen/ERα may function as an epigenetic switch for MSC adipogenic fate determination and modulate chromatin modifications coordinately.

Accumulated evidence highlighted the role of EZH2 in cell fate determination of MSC regulating adipogenic and osteogenic differentiation (Wang et al., 2010; Chen et al., 2011; Chen et al., 2016). However, it is not clear how the EZH2-mediated epigenetic silencing coordinates molecular events during differentiation. It was reported that the reciprocal action of EZH2 and KDM6A, which is an H3K27 demethylase, determines an epigenetic switch in MSC differentiation (Hemming et al., 2014). Wei and colleagues demonstrated that in human mesenchymal stem cells, CDK1 promotes mesenchymal stem cell differentiation into osteoblasts *via* phosphorylation of EZH2 at Thr 487 (Wei et al., 2010). We revealed that liganded-ERα facilitates H3K27 methylation on adipogenic target genes physically interacting with EZH2 in MSCs both in ovex and normal animals.

DNA methylation is a heritable modification that favours genomic integrity and ensures proper regulation of gene expression. It largely contributes to gene silencing (Zeng & Chen, 2019). We examined the DNA methylation status of PPARγ and C/EBPα upon estrogen administration. We have observed mosaicism in CpG methylation profile on adipogenic gene promoters between and within MSC clones similar to a previous study (Noer et al., 2006; Teven et al., 2011; Sinkkonen et al., 2008). Significantly higher DNA hypermethylation upon estrogen administration was only observed on C/EBPα in normal female-MSCs. On the contrary, there were no significant differences observed within ovex groups on C/EBPα and PPARγ.

In previous studies, the cross-talk between H3K27 methylation and DNA methylation was not demonstrated in stem cells. In normal cells, it is widely accepted that the genes which are repressed by EZH2 containing polycomb repressive complex 2 (PRC2), have mostly unmethylated CpG islands (Cedar & Bergman, 2009; Widschwendter et al., 2007). One possible explanation for this observation is that during gene silencing heterochromatinization is initiated by the polycomb repressive complexes. Subsequently, acquisition of DNA methylation occurs as a secondary event for long-term repression stability (Lande-Diner & Cedar, 2005). This was supported by a study which showed that *de novo* DNA methyltransferase three-like (Dnmt3L), which is a catalytically inactive DNA methyltransferase, interacts with the PRC2 complex in competition with the active DNA methyltransferases, Dnmt3a/3b. This maintains hypomethylation at the EZH2 and H3K27me3 positive promoters of the bivalent developmental genes in embryonic stem cells (Neri et al., 2013). Our bisulfite sequencing analyses were also demonstrated that estrogen has a role in the regulation of histone modifications, however, this interaction was not observed in DNA methylation in MSC. We concluded that estrogen reduces adipogenic gene transcription mainly acting on H3K27 methylation. This result also has been proved that in addition to methylation events, other epigenetic regulatory processes such as histone acetylation or recruitment of HMT complexes are also necessary to examine for the understanding underlining mechanism of differentiation of stem cells.

## Conclusions

**Figure 5:**
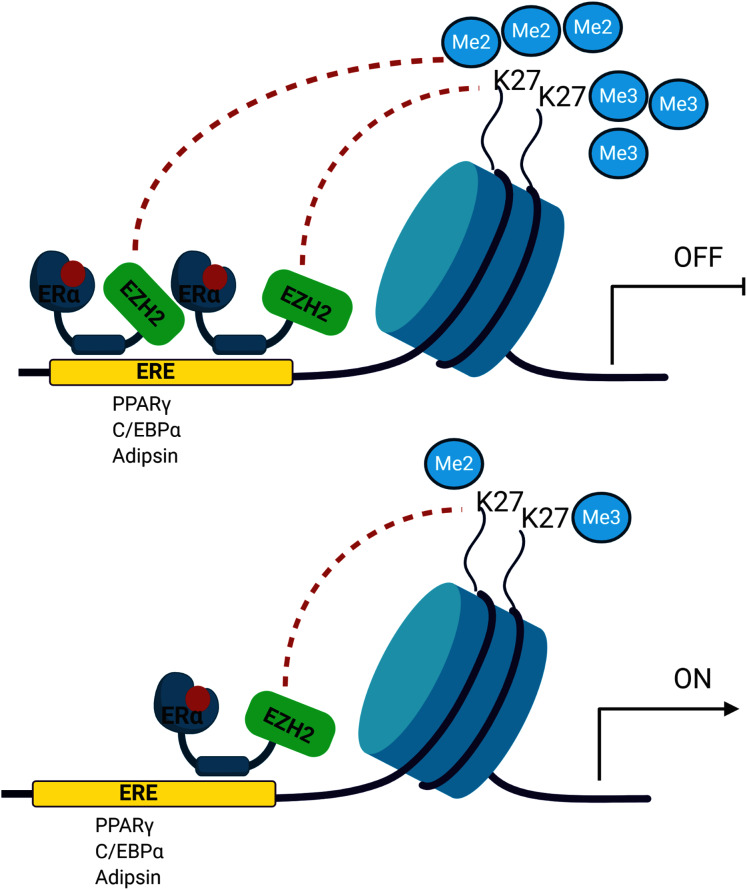
A schematic illustration of the regulatory role of estrogen in MSC cell fate. Ligand activated-ERα mediates H3K27 methylation of adipogenic transcription factor genes by directly interacting with EZH2. Increased binding of ERα may lead to enhance H3K27 methylation on the promoter region of PPARγ, C/EBPα and Adipsin. This in turn causes the transcriptional repression of PPARγ, C/EBPα and Adipsin upon estrogen treatment. On the other hand, in the absence of estrogen treatment, accumulation of methylated H3K27 on the adipogenic genes decreases as a result of reduction in binding of ERα to these genes. The decrease in H3K27 methylation causes transcriptional activation of adipogenic genes in MSCs. We suggest that estrogen may hinder MSC commitment into adipocytes by regulating epigenetic regulators. (The figure was designed with BioRender).

The signalling cross-talk between ERs and adipogenesis has been suggested in several studies. Our results may also bring an alternative explanation of how estrogen hormone and ERα inhibit adipogenesis in MSCs associated with epigenetic mediators. Our findings revealed that ERα induces enrichment of the H3K27 methylation on the promoters of master adipogenic transcription factor genes causing the inhibition of gene expression followed by attenuation of adipogenic differentiation in estrogen-dependent-manner in MSC. Estrogen may facilitate this methylation through modulating the interaction of ERα with EZH2 which is the H3K27 methyltransferase enzyme (Fig. 5). To date, only a few studies focusing on epigenetic regulation of MSC adipogenic differentiation have been conducted. An in-depth understanding of these mechanisms may provide findings to be easily translated into clinical practice given that the key role exerted by epigenetic deregulation in the development of obesity and determination of metabolic disorders has been widely acknowledged.

## Supplemental Information

10.7717/peerj.12071/supp-1 Supplemental Information 1 Changes in mRNA expression of the osteogenic transcription factor upon estrogen treatment in MSCs. The mRNA expression levels of RUNX2 gene in MSCs isolated from normal (female) and ovariectomized female (ovex) rats were cultured in the absence and presence of estrogen. Transcript levels were normalized to Cylclophilin. * indicates *p* < 0.05. All data are represented as the mean ± SD (*n* = 6)

10.7717/peerj.12071/supp-2 Supplemental Information 2 ARRIVE Checklist.

10.7717/peerj.12071/supp-3 Supplemental Information 3 Raw data for qRT-PCR.

10.7717/peerj.12071/supp-4 Supplemental Information 4 Western blot uncropped gel for Fig. 2.

10.7717/peerj.12071/supp-5 Supplemental Information 5 IP IgG control western gel raw data for Fig. 2.

10.7717/peerj.12071/supp-6 Supplemental Information 6 Raw data for the ChiP qPCR of Fig. 3.

## Additional Information and Declarations

### Data Availability

The following information was supplied regarding data availability: The raw measurements are available as Supplemental Files.
