# Supplementary figures and images for: Estrogen receptor alpha regulates the expression of adipogenic genes genetically and epigenetically in rat bone marrow-derived mesenchymal stem cells
Source: PeerJ. 2021 Sep 10;9:e12071. doi: 10.7717/peerj.12071 (PMC8436959; doi:10.7717/peerj.12071)

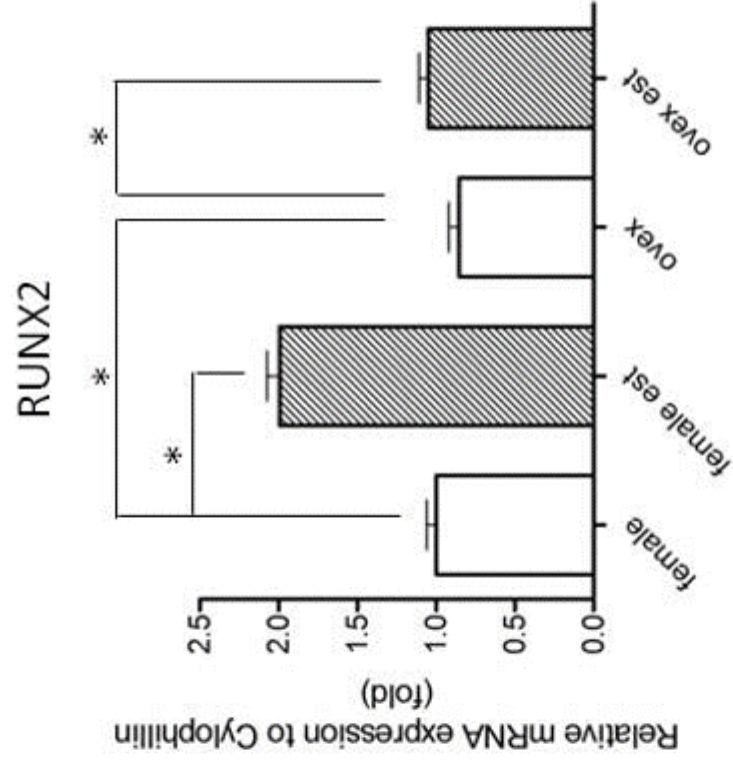

Supplement: Supplemental Information 1 — The mRNA expression levels of RUNX2 gene in MSCs isolated from normal (female) and ovariectomized female (ovex) rats were cultured in the absence and presence of estrogen. Transcript levels were normalized to Cylclophilin. * indicates p < 0.05. All data are represented as the mean ± SD (n = 6) [file peerj-09-12071-s001.pdf]

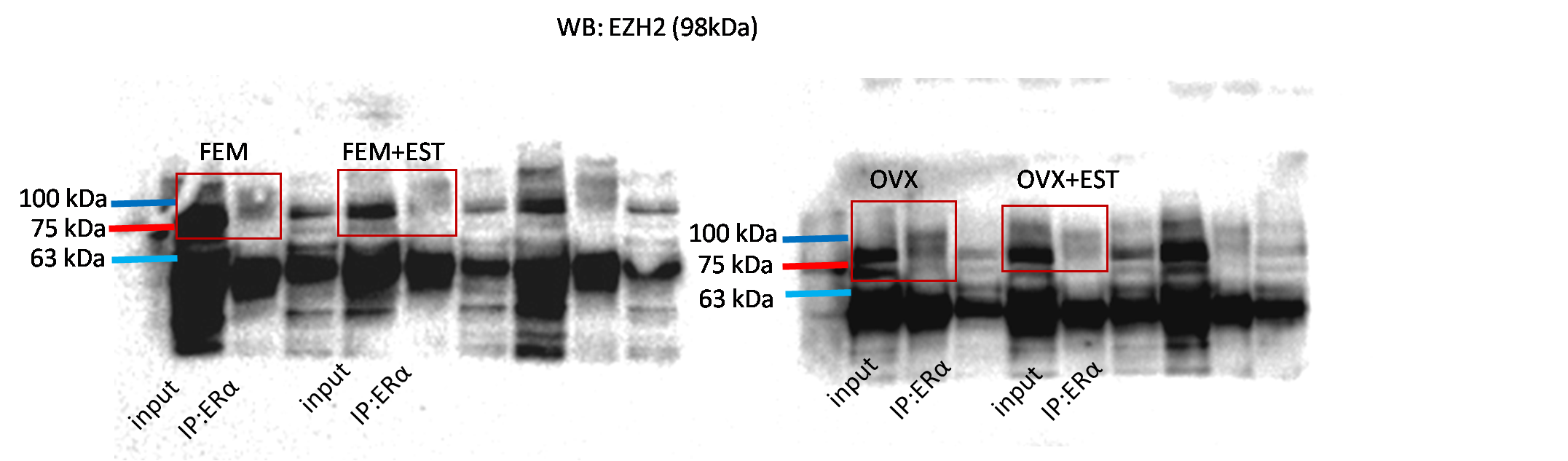

Supplement: Supplemental Information 4 [file peerj-09-12071-s004.png]

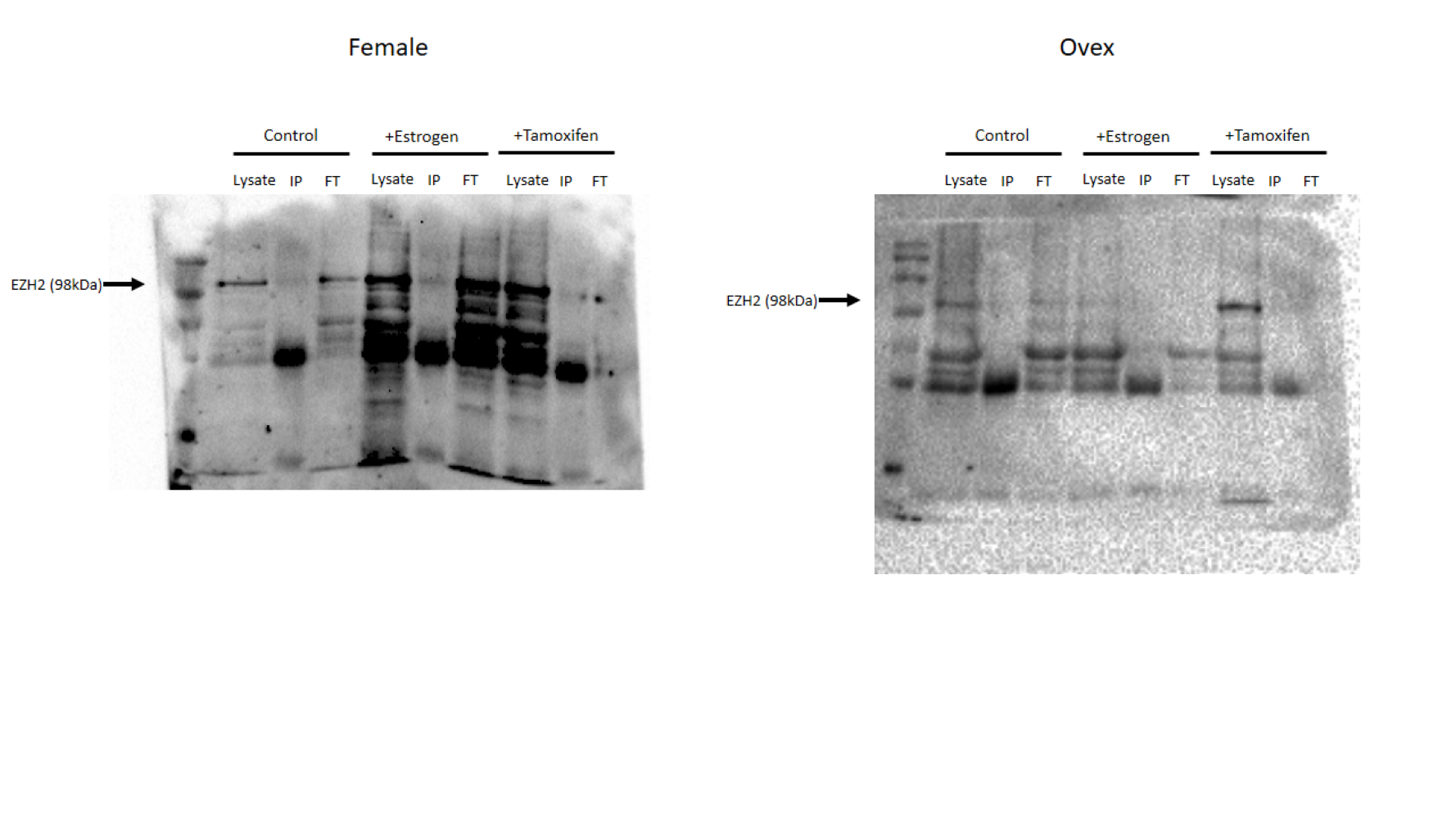

Supplement: Supplemental Information 5 [file peerj-09-12071-s005.png]
